# Microbial Consortium of *Streptomyces* spp. from Mining Environments Enhances Phytoremediation Potential of *Lemna minor* L.

**DOI:** 10.3390/plants14223467

**Published:** 2025-11-13

**Authors:** Rihab Djebaili, Beatrice Farda, Oscar Gialdini, Ilaria Vaccarelli, Younes Rezaee Danesh, Marika Pellegrini

**Affiliations:** 1 Department of Life, Health and Environmental Sciences, University of L’Aquila, Via Vetoio 1, 67100 L’Aquila, Italy; rihab.djebaili@univaq.it (R.D.); oscargiuseppe.gialdini@graduate.univaq.it (O.G.); marika.pellegrini@univaq.it (M.P.); 2 Molecular Ecology Group (MEG), Water Research Institute (IRSA), National Research Council (CNR), Corso Tonolli 50, 28922 Verbania, Italy; ilaria.vaccarelli@iusspavia.it; 3 Department of Plant Protection, Faculty of Agriculture, Van Yuzuncu Yil University, Van 65090, Türkiye; younesrezaeedanesh@yyu.edu.tr

**Keywords:** heavy metals, duckweed, metabarcoding, bioaugmentation, actinobacteria, phytoremediation

## Abstract

The presence of substantial amounts of heavy metals in the environment can result in various significant ecological issues and human health risks. Currently, bioremediation employing microorganisms is garnering significant interest due to its effectiveness. The present investigation aimed to isolate actinobacterial strains from an Italian mine and to characterise them for heavy metals resistance and plant growth-promoting characteristics. The different samples were processed for DNA extraction and 16S rRNA gene metabarcoding to investigate the bacteria and archaea communities. Cultivable microbiota were isolated and evaluated for heavy metals tolerance and different PGP traits. The most pertinent strains were tested for compatibility, merged into a consortium, and tested on *Lemna minor* L. Metabarcoding analysis revealed that amplicon sequence variants (ASVs) at the phylum level were mostly assigned to proteobacteria and bacteroidota. Uncultured and unknown taxa were the most prevalent in the samples at the genus level. A total of ten strains were obtained from the culture-dependent approach exhibiting interesting heavy metals tolerance and plant growth-promoting traits. The best strains (MTW 1 and MTW 5) were selected and further characterised by 16S barcoding. These strains were identified as *Streptomyces atratus* (99.57% identity). An in planta experiment showed that the metal-tolerant consortium MTW 1-5 improved plant physiology by significantly optimising plant growth and tolerance to heavy metals. The experiment conducted provided evidence for the possibility of using actinobacteria as bioaugmentation agents to improve the phytoextraction abilities of *L. minor*.

## 1. Introduction

One of the main environmental challenges is the disruption caused by the world’s rapid population growth [1]. This phenomenon further exacerbates the problems caused by rapid industrialisation, especially in developing countries, and the management of the consequent industrial waste products, with serious concerns regarding the improper disposal of pollutants and their dumping into water bodies (ponds, rivers, and streams) and land regions [2]. While some contaminating agents are easily handled, others are undegradable, such as heavy metals. Their build-up in the environment facilitates their dispersal through soil and water, endangering the survival ability of organisms exposed to them [3]. Heavy metals constitute the primary elements of the earth’s crust and are persistent environmental pollutants that cannot be completely decomposed by biological mechanisms. However, they can be converted into non-toxic forms [4,5]. From a chemical perspective, heavy metal designates elements and compounds possessing a particular gravity of above 5 and an atomic mass exceeding 20. Examples of heavy metals include arsenic (As), cadmium (Cd), copper (Cu), chromium (Cr), lead (Pb), mercury (Hg), nickel (Ni), and zinc (Zn). In biology, the term “heavy” denotes several metals, and occasionally metalloids, which might be detrimental to flora and fauna even in minimal doses [6]. Even if minimal amounts of these metals are fundamental in many enzymatic and metabolic processes, acting as cofactors, these metals can be highly toxic in excess to all living organisms [7].

Environmental pollution with heavy metals is countered by landfilling, involving the creation of cells typically made of cement or metal to enclose the pollutants with barriers [8]. However, this approach is costly and provides only short-term coverage. Moreover, continuing to execute these procedures has become less appealing due to the hazards associated with secondary pollution and workers’ safety. Other techniques that raise concerns about efficiency are ion exchange, solvent extraction, membrane separation, physicochemical separation, and other electrochemical and physicochemical techniques [9,10,11]. A sustainable approach involves bioremediation, mostly phytoremediation, an advanced cleaning approach emerging from this biotechnological field [9].

Among phytoremediation plants, *Lemna minor* L., also known as duckweed, is a pervasive floating aquatic macrophyte that has ecological and economic implications in areas where plant colonies are present [12]. Duckweed exhibits a wide distribution across several geographical locations, from freshwater to brackish water and from tropical to temperate regions [13]. Duckweed has been extensively utilised in aquaculture, animal husbandry, poultry production, biofuels, pharmaceuticals, toxicological assessments, environmental monitoring, and wastewater remediation [2,12,14,15]. Bacterial metal tolerance in polluted environments has been considered as essential for plant-associated bacteria [16]. It may also apply to plant metal uptake, as bacterial metal resistances could change the bioavailability of metals [17]. Bacteria would shield plants from harmful metals in the environment by creating a site of attachment and nutrient uptake [18]. Similarly, bacteria would shield plants from metal toxicity by expelling metals outside their cell walls [16]. Duckweed has also been found to recycle nutrients and has been utilised in ecotoxicological and phytoremediation research with related bacteria, especially members of the *Lemna* and *Spirodela* genera [19,20,21].

Phytoremediation can be improved with the application of bacteria via bioaugmentation or rhizoremediation [22]. Microorganisms, including bacteria, yeast, fungi, and archaea in association with plants, are crucial for remediating industrial disposals, such as pesticides, heavy metals, and toxic chemical fertilisers [3,23]. They act as biological catalysts in a bioremediation system [24], which is composed of appropriate components for the rehabilitation of contaminated environments. Microorganisms can adsorb and convert heavy metals, whereas plants are better suited for metal removal and immobilisation from soil [3]. The application of plants and their related microbes for the remediation of contaminated and degraded environments by heavy metals is a new field, offering merits in cost, sustainability, and environmental benefits [25,26].

Bacteria contribute to the growth and well-being of the host plant as well as the phytoremediation capacity to take up pollutants. Plant growth-promoting bacteria (PGPB) benefit the host plant in numerous ways, such as nitrogen fixation, phosphate solubilisation, siderophores and phytohormones production [27], detoxification, and pollution mitigation [21]. Most bacteria that are related to duckweed, known as duckweed-associated bacteria (DAB), prove to be PGPB for duckweeds [19]. Actinobacteria are Gram-positive, aerobic, sporulating, filamentous bacteria that occur at high densities in soil. Owing to their massive synthesis of enzymes and secondary metabolites, including immune suppressants, degrading enzymes, enzyme inhibitors, pesticides, insecticides, phytotoxins, and phytohormones, actinomycetes are becoming increasingly critical to investigators [28,29,30,31].

Actinobacteria plant stimulation is related to the generation of phytohormones, siderophores, atmospheric nitrogen fixation, phosphate solubilisation, and the inhibition of the formation of plants ethylene in stressful conditions using 1-amino cyclopropane-1-carboxylate (ACC) deaminase activity [30,31]. These microorganisms also have numerous features that make them ideal for the bioremediation of soils affected with organic and inorganic pollutants. They have a crucial function in recycling organic carbon and can break down intricate polymers through the secretion of external degrading enzymes and peroxidases [32,33]. Hence, the employment of metal-resistant actinomycetes, which have a symbiotic relationship with plants, holds significance as they can supply and make nutrients like iron (Fe) and phosphorus (P) available to plants. These, in turn, can neutralise the heavy metals’ toxic effects [28]. Due to the high amount of metal and mineral deposits and the prevalent contamination frequently caused by mines, mining areas are suitable locations for heavy metal-tolerant bacteria isolation. We assumed that mines could be a promising source of obtaining actinobacteria that are useful in creating a bioaugmentation consortium. The present investigation aimed to isolate actinobacteria from a mine location and to examine their tolerance to heavy metals (HMs) and plant growth-promoting (PGP) traits on duckweed. DNA extraction and 16S rRNA gene metabarcoding were conducted on the different samples to examine the bacteria and archaea communities. Cultivable microbiota were isolated and tested for their tolerance to heavy metals and several PGP features. The most suitable strains were evaluated for compatibility and mixed in a consortium. The bioaugmentation ability of the latter was tested on duckweed under a controlled environment.

## 2. Results

### 2.1. DNA Extraction and 16S rRNA Metabarcoding

The metabarcoding results of the 16S rRNA gene analysis were utilised to evaluate the samples’ diversity (Table 1).

The LAK and MIN samples presented more taxa numbers (1273 and 1036) and total reads (28,505 and 18,070) compared to the MTW sample (taxa = 740 and total reads = 13,029). All the samples exhibited significant diversity and species richness (Shannon_H’ index higher than 6 and Chao-1 greater than 700) accompanied by a high degree of homogeneity within the community (Simpson 1-D close to 1). A moderate evenness score was observed, suggesting a microbial community diversification fairly homogeneous with regard to the main taxa.

**Figure 1:**
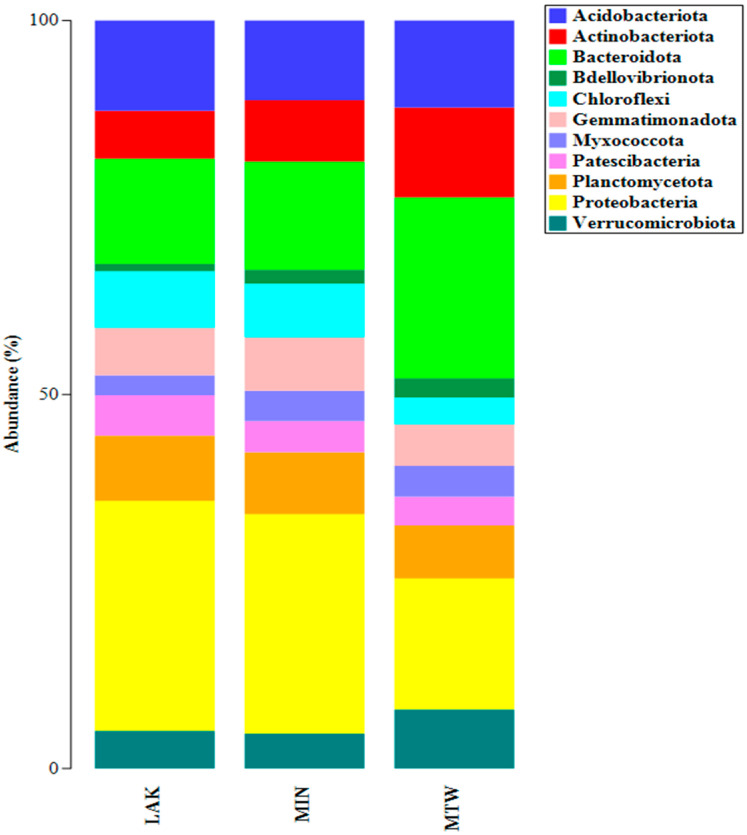
Taxonomic bar plot of the relative abundances of bacterial phyla associated with individual soil samples.

To analyse the ASVs composition at various taxonomic levels, data were filtered by applying a relative abundance threshold of 2% and displayed using taxonomic bar plots. Figure 1 demonstrates the distribution of ASVs at the phylum level. The ASVs were mostly associated with proteobacteria (syn. Pseudomonadota) (30% of relative abundance on average for the MIN and LAK samples and 18% for the MTW sample) and *bacteroidota* (syn. *Bacteroidetes*) (24% of relative abundance for the MTW sample and 14% of relative abundance on average for the MIN and LAK samples), followed by *Actinobacteriota* (syn. *Actinomycetota*) for the MTW sample with 12% relative abundance. *Acidobacteriota* (11% of relative abundance) were evenly distributed across all samples. Other phyla, *Planctomycetota* (syn. *Planctomycetes*), *Chloroflexi* (syn. *Chloroflexota*), *Verrucomicrobiota*, and *Gemmatimonadota,* were moderately abundant in all samples.

**Figure 2:**
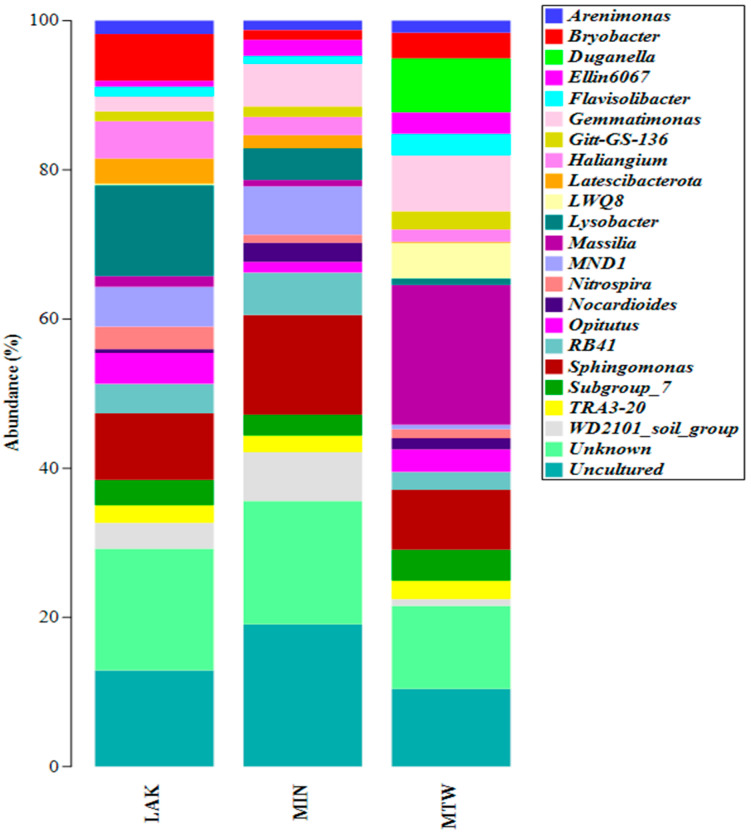
Taxonomic bar plot of the relative abundances of bacterial genera associated with individual soil samples.

The ASVs were associated with 23 taxa (2% cut-off) at the genus level (Figure 2). Taxa labelled as Uncultured and Unknown—indicating sequences not confidently classified at the genus level—were the most prevalent in the MIN and LAK samples (19% and 16%, respectively), while *Massilia* was dominant in the MTW sample (19%). Unknown taxa were the second-most-abundant group in MTW and MIN, while Uncultured ranked second in LAK. *Sphingomonas* was relatively abundant across all samples, whereas *Lysobacter* was found only in LAK samples (12%) and *Duganella* and LWQ8 were exclusively found in MTW samples. *Gemmatimonas* was consistently detected at moderate abundance in all samples.

### 2.2. Actinomycetes Isolation and In Vitro Heavy Metals Tolerance

Actinomycetes strain isolation and purification on ISP_2_ culture medium resulted in ten strains with different morphologies: two from the internal area (MIN 1 and MIN 2) and eight from the twilight zone near the entrance (MTW 1, MTW 2, MTW 3, MTW 4, MTW 5, MTW 6, MTW 7, and MTW 8). After a purity check, the strains’ capacity to withstand heavy metals was assessed by examining bacterial growth with various metal concentrations (Al, Cd, Cu, Mn, Ni, Pb, and Zn). The results are shown in Table 2 below.

**Figure 3:**
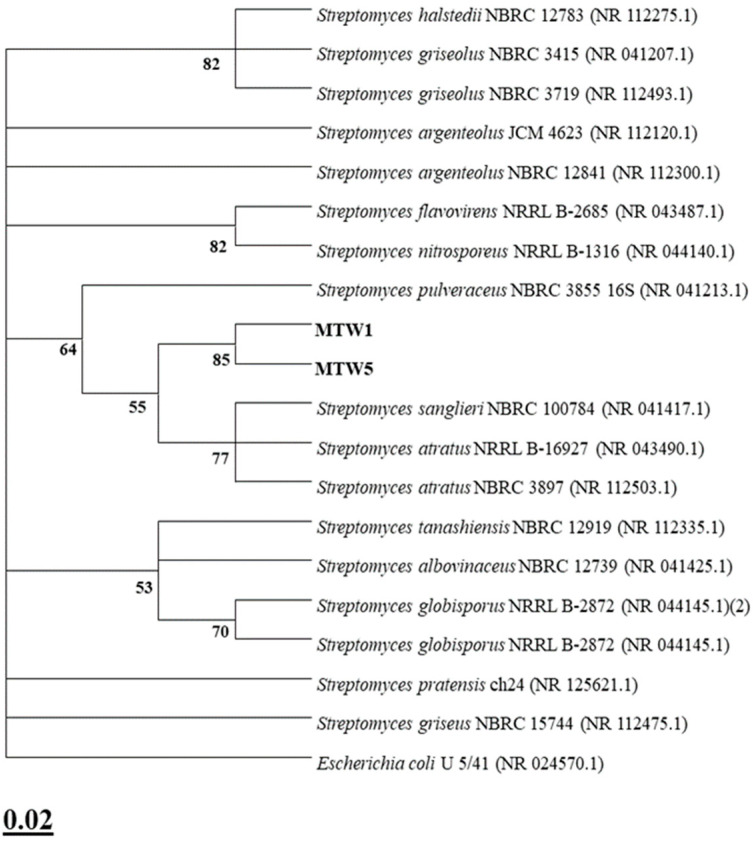
Phylogenetic analysis of actinomycetes isolates (MTW 1 and MTW 5) compared with the closest species of the genus *Streptomyces*. (GenBank accession numbers are shown in brackets). The maximum likelihood method was used with a bootstrap consensus tree (from 1000 replicates to represent the distance). *Escherichia coli* U 5/41 was introduced as an outgroup. The number of substitutions per nucleotide site for a unit of branch length is given in the scale bar (0.02).

Based on these results, two strains (MTW 1 and MTW 5) were selected and further characterised through 16S rRNA gene barcoding. The isolates were associated with the *Streptomyces* genus through phylogenetic analysis. Isolates MTW 1 and MTW 5 were identified as *Streptomyces atratus* (99.57% identity). Figure 3 displays the strains’ phylogenetic position.

### 2.3. Plant Growth-Promoting Activities

The results of the plant growth-promoting activities of the isolates are shown in the table below (Table 3).

Phosphate solubilisation ability was expressed by four strains among the all the strains assessed, with the highest value obtained by the MTW 1 isolate (13.37 µg mL^−1^), which was the only producer for indoles (2.85 µg mL^−1^). Almost all strains expressed siderophore production (70%); the MTW 3 strain obtained the highest value (24.95%), followed by MTW 5 and MTW 1 (22.20% on average). The lowest value was observed for the MIN 2 strain (13.15%). All the strains expressed hydrogen cyanide and ammonia production with various intensities depending on the strain. The MTW 6 strain showed high HCN production, although high NH_3_ production was also signalled by strains MTW 5, MTW 7, and MIN 1. The isolated strains had no ACC deaminase activity.

### 2.4. In Planta Experiment on Lemna minor L.

During the in planta experiment, plants inoculated with the consortium (PGPA) enhanced duckweed growth in normal conditions and under heavy metals stress relative to the control (untreated plants) (*p* < 0.05). The plants’ morpho-biometric parameters are presented in Table 4 below. Plant biomass was slightly higher in the plants treated with PGPA compared to the control; however, this difference was not statistically significant (*p* > 0.05). Consistent patterns were reported under HMs stress, and inoculation with actinomycetes strains resulted in increased plant biomass respective to the control, although the difference was not statistically significant (*p* > 0.05). Regarding root lengths, inoculation with the consortium resulted in slight increases in plant lengths under normal conditions (*p* > 0.05), and with HM stress, a moderate difference in root lengths was observed but the difference was not statistically significant (*p* > 0.05).

**Figure 4:**
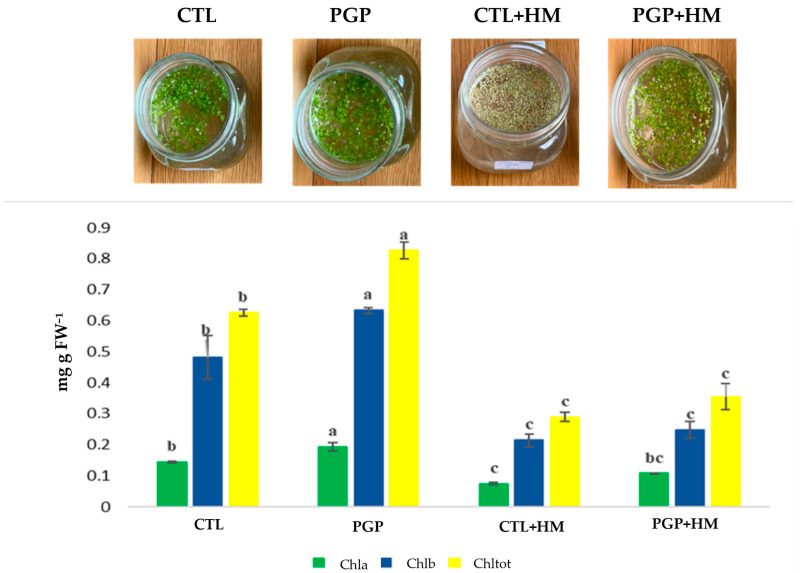
Contents of chlorophyll a (Chla), chlorophyll b (Chlb), and total chlorophyll content (Chltot) in *Lemna minor* L. uninoculated (CTL) and inoculated (PGP) with the consortium in normal conditions and under heavy metals (HMs) stress (CTL + HM: uninoculated plants with heavy metals stress, PGP + HM: inoculated plants with heavy metals stress). Results followed by the same case letters are not significantly different according to Tukey’s post hoc test (*p* < 0.05).

Inoculation with actinomycetes strains enhanced the chlorophyll content of duckweed in comparison with the untreated plants (*p* < 0.05). The concentrations of chlorophyll a, b, and total contents are presented in the figure below (Figure 4). In the absence of HMs stress, plants inoculated with the consortium had higher chlorophyll a, b, and total contents (Chltot = 0.83 mg g FW^−1^) in contrast to the uninoculated plants (*p* < 0.05). Under HMs stress, a difference in chlorophylls content was obtained, with higher content observed in treated plants (*p* > 0.05), but this difference was not statistically significant.

## 3. Discussion

The metabarcoding results indicated that the prevalent amplicon sequence variants (ASVs) were linked to proteobacteria, bacteroidota, and actinobacteriota at the phylum level. Proteobacteria (syn. Pseudomonadota) presented 30% of relative abundance on average for the MIN and LAK samples and 18% for the MTW sample. Proteobacteria are one of the largest prokaryote divisions, accounting for most of the known Gram-negative bacteria [34,35,36]. The term proteobacteria was first suggested by Stackebrandt et al. [37]. The taxonomic origin of the phylum derives from the ‘purple bacteria,’ comprising four distinct bacterial groups characterised by their 16S rRNA gene sequence structures [38,39]. The proteobacteria phylum is classified into six classes, as established by the 16S rRNA gene’s phylogenetic analysis. These classes were previously considered as subdivisions of the phylum. Given that the classes are divided based on molecular relation, it is not surprising that members of each class lack distinct morphological or physiological features [36,40,41]. The cytophaga–bacteroides–flavobacterium phylum [38], known as bacteroidetes [42], consists of various bacterial species that exhibit extraordinary phenotypic variety. This phylum showed 24% of relative abundance for the MTW sample and 14% of relative abundance on average for the MIN and LAK samples. These bacteria can thrive in different environments, displaying aerobic and anaerobic lifestyles [43]. Bacteroidetes are relatively unexplored regarding phylogenetic diversity [44,45]. Although environmental studies have demonstrated initial glimpses into the genetic potential of the phylum [46], no comprehensive assessment into their genetic and metabolic roles has been conducted. In addition, there are still only a few isolated compounds from this group [47]. Bacteroidetes are one of the 20 important phyla comprising eubacteria [42]. Its branching position has been determined by the examination of protein signature sequences and is located between the proteobacteria and spirochetes [43]. The actinobacteria phylum is one of the most significant taxonomic classifications of the 18 major known lineages in the bacteria domain, in terms of quantity and species diversity [48,49]. It displayed 12% of relative abundance for the MTW sample. This phylum comprises Gram-positive bacteria with enormous genome contents of guanine (G) and cytosine (C). Phylogenetic analysis through 16S rRNA sequences showed that actinobacteria are classified into 6 classes, 79 families holding 46 orders and 10 new families holding 16 new orders [48,50]. These bacteria exhibit great form diversity, from coccoid (e.g., *Micrococcus* spp.) or rod-coccoid (*Arthrobacter* spp.) to fragmenting hyphal types (*Nocardia* spp.) or permanent and well-defined branched mycelium (*Streptomyces* spp.) [51]. They also exhibit diversity in their metabolic and physiological characteristics, including extracellular enzyme production and a vast range of secondary compounds [52]. The majority of these secondary metabolites exhibit antibacterial properties [53]. Actinobacteria exhibit typical characteristics. They exist in various ecosystems, including terrestrial and aquatic environments, as well as endophytically within plants. Actinobacteria species are present in standard, particular, and extreme habitats with temperature variations, elevated radiation, alkaline or acidic pH, salinity, and low humidity and nutrient availability [54]. The samples presented an equal distribution of *acidobacteriota* (11% relative abundance). *Acidobacteriota* is a widespread bacterial phylum present in different ecosystems, including soils, marine environments, and freshwaters. The phylum was first identified in 1991 when *Acidobacterium capsulatum* was found in an acidic mineral environment [55]. Subsequently, the broad distribution of the phylum was unveiled by identifying its distinctive 16S rRNA gene sequence. These microbes’ abundance and ubiquity point to concerns linked to their ecological role and metabolism [56]. Initially described in 1997 with only three cultivated members [57,58], *Acidobacteriota* has evolved into a diverse phylum. It is categorised into 15 class-level categories, with just 5 possessing the 62 successfully cultured and well-documented *acidobacteriota* [59]. The 16S rRNA gene sequencing tools have shown that this phylum can account for up to half of the overall bacterial community, with an overall contribution of approximately 10–20% of the soil bacteria worldwide [57,60]. Ecological surveys of several ecosystems have shown that *acidobacteriota* is not limited to soil environments. It also exists in other marine and extreme settings, like acid mine drainage systems and hot springs [61]. As microorganisms, they are a significant constituent of the terrestrial ecosystem and are important for the maintenance of ecological services [57].

The ASVs were associated with 23 taxa at the genus level (2% cut-off), with Uncultured and Unknown as the most dominant taxa in the MIN and LAK samples (19% and 16%, respectively). Unknown taxa were the second-most-abundant group in MTW and MIN, while Uncultured ranked second in LAK. Over 99% of the bacteria found in natural settings have not been cultured yet [62,63]. They represent a black box containing a latent population with an unexplored genetic reservoir containing unique and important catalysts, enzymes, and precursors for industrial and medical uses [63,64]. Despite the predominance of uncultivated bacterial and archaeal species on Earth, the advent of next-generation sequencing allowed direct access to their genomic information [65]. The proportion of ambient genes, henceforth termed “unknown,” lacking homologues in cultivated species might vary from approximately 25% [66] to more than 50% [67], contingent upon the methodology and particular context. This means that millions of genes with operational roles and ecological and evolutionary relevance remain uncharacterised [68]. Uncultivated species are prevalent in various forms of non-human environments and are regarded to perform important ecological functions; hence, studying their biology is critical to understanding how these ecosystems are affected [69]. Whereas sequencing technologies have greatly improved our comprehension of microorganisms’ diversity and function, acquiring cultured members of essential uncultured lineages is vital for the direct assessment of their physiological and metabolic functions, as well as enhancing our comprehension of their biology and ecology in natural settings [69]. It is estimated that 2.3 × 10^29^ (81%) and 2.2 × 10^29^ (25%) of the microbial cells in the Earth’s microbiomes belong to uncultured genera and phyla, respectively [70]. Non-cultivatable microorganisms are ubiquitous in diverse settings. They are crucial in the breakdown of several pollutants. They reflect a hidden population that harbours a genetic reservoir that encodes unique and valuable functions [71]. Recent studies have confirmed that uncultured bacteria may have a substantial role in environmental pollutant biodegradation [72].

*Sphingomonas* was relatively abundant in all samples and *Lysobacter* was relatively abundant in the LAK samples (12%). The *Sphingomonas* genus includes Gram-negative bacteria classified within the α-Proteobacteria clade. Distinctively, its cell wall contains sphingoglycolipids instead of lipopolysaccharides [73,74]. The pigment nostoxanthin imparts a yellowish or pink hue to their colonies on agar media [73,75]. In this context, especially in *Sphingomonas*, the precise colouration remains unknown and may be ascribed to other carotenoids or lycopenes [76]. These microorganisms can break down aromatic pollutants, making them excellent bioremediators [73]. *Sphingomonas* species exhibit diverse functions, such as the bioremediation of environmental pollutants and the synthesis of phytohormones. Several studies have revealed the degradation ability of organometallic compounds of *Sphingomonas*. However, further assessment is needed to define their real genus biotechnological effects. Certain members of the genus could induce plant growth under high-stress circumstances such as salinity, drought, and HMs contamination in agriculture [77,78,79]. This activity is associated with their capacity to release plant growth hormones [78]. Christensen and Cook [80] proposed that the genus *Lysobacter*, etymologically originated from “the lysing rod,” belongs to Xanthomonadaceae family of the Gammaproteobacteria class. *Lysobacter enzymogenes* presents the type species, later refined by Park et al. [81]. It is Gram-stain-negative, non-fruiting, gliding motile, aerobic, and has a high DNA G + C content [82]. *Lysobacter* strains are found in diverse habitats [82]. Members of the *Lysobacter* genus have been isolated from numerous aquatic and terrestrial ecosystems, including sludge, seawater, and deep-sea sponges, as well as from plant rhizospheres, plant-cultivated soils, caves, and lakes [83,84,85,86,87,88,89]. This genus’ members exhibit a lytic potency against a broad spectrum of microorganisms like nematodes, actinomycetes, cyanobacteria, green algae, yeasts, filamentous fungi, oomycetes, and Gram-negative and Gram-positive bacteria. They also actively degrade many polysaccharides like chitin [35,80]. The lytic processes are ascribed to the release of numerous extracellular enzymes, such as protease, endopeptidase, *glucanase*, lipase, and chitinase [35]. *Duganella* was present only in the MTW samples. The first member of the genus *Duganella* was discovered in wastewater in 1968 and designated as *Zoogloea ramigera* [90]. The taxonomic revision of *Zoogloea ramigera* resulted in the creation of the genus *Duganella* by Hiraishi et al. [91], with *Duganella zoogloeoides* designated as a unique species type. At the phylogenetic level, it is grouped in the Betaproteobacteria class, of the family *Oxalobacteraceae*. Nevertheless, the 16S rRNA gene analysis showed that the genus is polyphyletic [92,93]. *Gemmatimonas* were common and moderately present in all samples. Members of *Gemmatimonadota* phylum (formerly *Gemmatimonadetes*) are commonly found in soils, wastewater, biofilms, and plant-associated environments [94,95], ranking among the soils’ nine dominant phyla [94,96,97]. Diversity investigations using 16S rRNA genes disclose that *Gemmatimonadota* are highly suited to dry and oligotrophic environments [94]. Notwithstanding their extensive environmental distribution, the ecology, physiology, and environmental roles of *Gemmatimonadota* remain poorly comprehended [94,98].

*Streptomyces* strains used in this investigation displayed different plant growth-promoting properties and tolerance to different heavy metals concentrations. The experiment in planta showed a good effect of the consortium in enhancing plant growth attributes (survival rate, biomass, root lengths, and chlorophyll contents). The enhancement of *Lemna minor* L. (duckweed) growth was linked to its interaction with the *Streptomyces* species. Because chlorophylls are a limiting element in photosynthesis and inoculation with the consortium increased chlorophyll production, duckweed growth promotion may be achieved in part by boosting photosynthesis, as described by Yoneda et al. [99]. Duckweed has demonstrated a remarkable efficacy in the phytoextraction of organic materials, dispersed particles, soluble salts, and heavy metals from wastewater [100]. According to multiple studies, aquatic plants are appropriate for the elimination of heavy metals [101,102,103,104]. Duckweed-associated actinobacteria are coexisting microbes that influence duckweed growth and adaptation [105]. *Streptomyces*, a genus of actinobacteria, is renowned for its ability to synthesise a varied range of secondary metabolites, encompassing antibiotics, plant growth-promoting compounds, and bioactive chemicals [29,30,106]. They can be used to form symbiotic relationships with plants, for example, duckweed, by providing increased nutrition [99].

Overall, the growth and stress tolerance of PGPB-treated duckweed under HMs stress confirmed that the selected consortium possesses interesting potential for bioaugmentation. Actinobacteria have been studied extensively for their HMs bioremediation characteristics [107,108,109]. *Streptomyces* species were found to have excellent bioaugmentation characteristics for HMs remediation [110]. These microbes can degrade such pollutants effectively owing to their diminutive size and elevated surface-to-volume ratio, which offers them a large surface area for the interaction with HMs-contaminated matrices [111]. An in planta experiment revealed that the metal-tolerant consortium MTW 1-5 exhibited a significant bioaugmentation effect, as evidenced by the enhanced growth of *L. minor* under both normal and HMs-contaminated conditions. The enhanced growth of duckweed can be ascribed to enhanced nutrient uptake and root architecture that, in combination, enable pollutant and plant nutrient uptake. The capacity of strains to synthesise siderophores has been used in bioremediation processes, where siderophore-producing bacteria are used to aid in HMs-contaminated site detoxification through metal chelation [112,113,114]. Reports indicate that siderophore production has increased following treatment with various toxic HMs, indicative of their function in supporting microbial metal tolerance [115]. The increased biomass and health of *L. minor* with inoculum point toward phytohormone production, encompassing IAA, which reduces stress impacts and promotes growth [116]. These characteristics are typical of actinobacteria such as *Streptomyces* spp., which are renowned for their tolerance in metal-contaminated habitats.

Different studies have emphasised the advantageous function of PGPB in raising the efficiency of HMs phytoremediation in hyperaccumulator plants. Babu et al. [117] investigated the isolation and identification of endophytic bacteria from *Pinus sylvestris* roots, resulting in the isolation of *Bacillus thuringiensis* GDB-1. The bacterium possessed some PGP properties and enhanced the phytoremediation capability of the hyperaccumulator plant *Alnus firma*. The findings show that the inoculation of *A. firma* with *B. Thuringiensins* GDB-1 enhances its phytoremediation effectiveness in heavy metal-polluted soil in mine tailings. Equally, co-culturing an hyperaccumulator plant, *Solanum nigrum*, and a heavy metal-tolerant PGPB (HMT-PGPB), *Bacillus* sp. PGP15, facilitate phytoremediation of cadmium-contaminated soil by stimulating plant growth, metal sequestration, and resistance to oxidative stress [118]. Four new aquatic PGP strains, MRB1-MRB4, were isolated from the *Lythrum anceps* rhizosphere [119]. Co-culturing with *L. minor* resulted in a 2.1- to 3.8-fold stimulation of frond numbers after 14 days, along with increased plant biomass and chlorophyll content. Actinobacteria boost rhizosphere colonisation, leading to the increased transfer of higher metals from water to the plant tissue [107]. Several research studies have validated the efficacy of bioaugmentation in HMs-polluted soils. Mahbub et al. [120] highlighted the decontamination of artificially polluted soil with Hg by *Sphingobium* SA2 with a 50% clearance efficiency. Ibarrolaza et al. [121] reported the decontamination of artificially hexavalent chromium (Cr (VI))-contaminated soil by *Sphingomonas paucimobilis* with a 90% removal efficacy. It has been reported that the (Cr (VI)) removal from artificially contaminated soil applying a mixture of *Streptomyces* sp. M7, *Streptomyces* sp. MC1, *Streptomyces* sp. A5, and *Amycolatopsis tucumanensis* achieves a removal effectiveness of 86% [122]. These bacteria may efficiently eliminate HMs because of their diminutive size and elevated surface-to-volume ratio, which affords them an extensive surface area for interaction with heavy metal-contaminated environments.

## 4. Materials and Methods

### 4.1. Sampling Area

**Figure 5:**
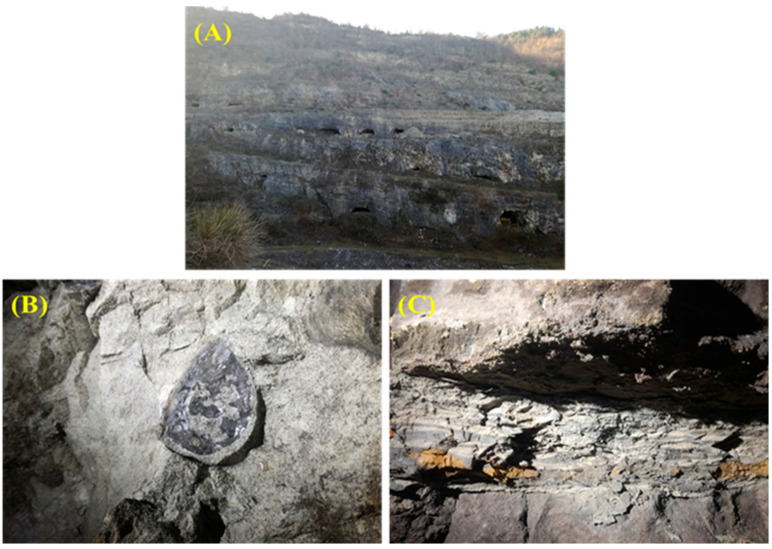
(**A**): Exterior of the Valle mining Romana site in the municipality of Manoppello (PE) in Majella mining; (**B**): internal area (MIN); and (**C**): area near the entrance (MTW).

Sampling occurred in April 2020 at the Foce Valle mining Romana site, *Manoppello* (PE) municipality, in the northern Maiella mining district (42°04′34″ N, 14°05′21″ E) (Figure 5). Specifically, the site under investigation is in the tertiary carbonate succession outcropping in the crest zone of the Maiella anticline (central Apennines) at about 400 m above sea level. Site-specific heavy metal concentration data is not available in the accessible literature. However, a regional environmental report for the Abruzzo region highlights the contamination of soils and groundwater by heavy metals, including arsenic, selenium, lead, and cadmium, in this area [123].

The mine consists of an open pit quarry and a complex system of tunnels distributed along eight levels (hypogean environment) communicating with the quarry itself. Samples were taken from different points (Figure 5): the lake in the lower portion of the cave (LAK) (Figure 5A) and from two large areas, an innermost zone (MIN) (Figure 5B)—characterised by rounded formations indicative of microbial action—and a twilight zone near the inlet (MTW) (Figure 5C), where there was corroded material typical of microorganism activity. The samples were kept refrigerated and transported to the laboratory. For the cultural approach, three subsamples from each specimen were processed individually. To conduct the molecular analysis, five aliquots from each specimen were mixed and placed into a solution of RNAlater (Ambion, Austin, TX, USA) following the manufacturer’s recommendations. All specimens were maintained at −80 °C until analysis.

### 4.2. DNA Extraction and 16S rRNA Gene Metabarcoding

The DNA extraction was carried out on the different homogeneous samples (500 mg), referring to the manufacturer’s procedure (NucleoSpin^®^Soil, Macherey Nagel, Germany). The purity and concentration of the DNA were checked utilising a Nanodrop spectrophotometer (Thermo Scientific^TM^, Waltham, MA, USA) and a Qubit fluorometer (Thermo Scientific^TM^, Waltham, MA, USA). From each sample, the separate replicates were merged in an equimolar proportion. A paired-end 16S rRNA community sequencing analysis was carried out through the Mi-Seq Illumina technology (Bio–Fab Investigation, Rome, Italy), emphasising the V3 and V4 regions of the 16S rRNA gene [124]. Following filtration, the sequencing reads were assessed for reliability and quantified. The DADA2 module was utilised to establish the ASV (amplicon sequence variant) with QIIME2 (qiime2–2020.2 version) [125]. The fit–classifier–naive–Bayes add-on module trained the classifier on the V3-V4 specific section of the 16S file sequences from the SILVA database (https://www.arb-silva.de/; accessed on 14 June 2025).

### 4.3. Isolation and Identification of Actinomycetes Strains

Actinomycetes strain isolation was carried out on the International *Streptomyces* Project N° 2 (ISP_2_) culture medium employing the suspension–dilution approach [126]. Each sample (1 g) was suspended in 9 mL of sterile saline solution (0.9%) and vortexed vigorously. Serial dilutions were carried out (up to a 10^−4^ dilution). From each dilution, 0.1 mL was distributed on the surface of ISP_2_ culture medium. To prevent bacterial and fungal growth, amphotericin B (25 µg mL^−1^) and nalidixic acid (75 µg mL^−1^) were supplied to the culture media. Plates were put in the incubator at 30 ^◦^C until all colonies were fully developed. Following purity assessments, the acquired isolates were subsequently characterised, and bacteriological properties were examined using qualitative laboratory techniques (utilising reagents and colonies colouration) [127].

### 4.4. In Vitro Heavy Metal Tolerance

The tolerance for heavy metals of the isolates was evaluated using ISP_2_ agar medium supplemented with different HM concentrations. The metals examined were aluminium (Al), cadmium (Cd), copper (Cu), lead (Pb), manganese (Mn), nickel (Ni), and zinc (Zn). The concentration of each heavy metal is shown in the table below (Table 5) [128]. Mn-oxide-solubilising capacity was assessed on manganese basal medium (MMB) containing MnO_2_ [129]. Plates were microaerophilically incubated at 30 °C in anaerobic containers until visible colonies developed utilising anaerobic generation kits (Oxoid, Basingstoke, UK).

### 4.5. Plant Growth-Promoting (PGP) Activities

#### 4.5.1. Phosphate Solubilisation

The strains’ ability to solubilise phosphate was tested on liquid NBRIP (National Botanical Research Institute Growth) medium [130]. Pre-cultures on ISP_2_ medium were prepared and used to inoculate liquid NBRIP medium [30]. Following incubation, cultures were centrifuged [30,131], and the solubilised phosphorus was quantified utilising the Olsen and Sommers colorimetric technique [132], and results were reported as µg PO_4_^3^ mL^−1^.

#### 4.5.2. Indoles Production

The different bacterial strains were grown on liquid ISP_2_ with the addition of tryptophan (0.2% *w*/*v*). The cultures were incubated for seven days at 30 °C with agitation. Following that, they were centrifugated for 20 min at 3000 rpm [30,31]. Subsequently, 4 mL of Salkowski’s reagent was added to 1 mL of supernatant [133]. After incubation for 30 min in the obscurity, the mixture’s optical density was determined at 530 nm. IAA (Sigma, St. Louis, MO, USA) was utilised as a standard (y = 0.0089x + 0.0113; R^2^ = 0.9975) and the results were reported as μg IAA mL^−1^.

#### 4.5.3. Siderophore Production

The siderophore-producing capacity of actinomycetes isolates was evaluated utilising the universal CAS test [134]. For CAS preparation, 121 mg of CAS was dissolved in 100 mL of demineralised water and 20 mL of a solution made from 1 mM ferric chloride (FeCl_3_ 6H_2_O) prepared in 10 mM HCl. While stirring, the mixture was introduced into 20 mL hexadecyl trimethyl ammonium bromide (HDTMA). HDTMA solution was made by dissolving 729 mg of HDTMA in 400 mL of distilled water. Before further usage, the solution CAS-HDTMA was sterilised. From each single bacterial culture grown on liquid ISP_2_ medium, 100 µL of supernatant was introduced to separate wells of a microtiter plate (Sigma). Subsequently, CAS reagent was added (100 µL). Following incubation, the optical density of the mixture was determined at 630 nm via a spectrophotometer (SPEKOL 1300 UV VIS spectrophotometer (Analytik Jena, Jena, Germany). Each strain was replicated three times in a 96-well microplate, and the siderophore was determined as follows (Ar = the control absorbance and As = the sample absorbance):

$$Percent\;Siderophore\;Unit\;\left(PSU\right)=\frac{\left(Ar-AS\right)\times 100}{Ar}$$

#### 4.5.4. Ammonia and HCN Production

The hydrocyanic acid (HCN) production was quantified in a trypticase soy agar (TSA) medium supplied with glycine at a concentration of 4.4 g L^−1^. A Whatman-type filter, having an identical diameter as the Petri dishes, was immersed with 0.5% picric acid and 2% sodium carbonate solutions and then inserted into the lid of each inoculated Petri dish. The plates were sealed with parafilm and placed in the incubator for seven days at 30 °C. The change in colour from yellow to orange or brown indicated a positive result for HCN production [135,136]. Peptone water (PW) was used for ammonia (NH_3_) testing. Each isolate was inoculated with a suspension of spores (100 µL) in 10 mL of PW and underwent incubation for 7 days at 30 °C. Afterward, Nessler reagent (0.5 mL) was introduced to each tube. The formation of a yellow colour was considered a positive result [127].

#### 4.5.5. The 1-Aminocyclopropane-1-Carboxylate (ACC) Deaminase Activity

The estimation of the strains’ ACC deaminase activity was performed following the protocol of Brígido et al. (2015) [137]. The isolates’ spore suspensions were introduced into liquid ISP_2_ medium and shaken moderately for three days at 30 °C. Pellets were recuperated by centrifugation and washed using DF (Dworkin and Foster) salt minimum medium, devoid of a nitrogen source [138]. Subsequently, they were cultured for three days at 30 °C in DF salt minimum medium with 3 mM of ACC. After that, cultures underwent centrifugation and washing with 5 mL of 0.1 M Tris-HCl (pH 7.6). The suspended cells were collected into 1.5 mL microcentrifuge tubes. Following centrifugation, pellets were used for the enzymatic activity test. Pellets were suspended in 20 µL of toluene and 400 µL of 0.1 M Tris HCl (pH 8.0). Furthermore, cell lysate (50 µL) from each strain was allocated into microtubes with 5 µL of ACC (0.5 M). Following a 30 min incubation at 30 °C, 500 µL of 0.56 M HCl was added to each tube. After agitation, supernatant was obtained by centrifugation. A solution of α-ketobutyrate (sigma) in 0.1 M TRIS-HCl (pH 8.0) was utilised as the standard. The absorbance of the reaction mixture was assessed at 540 nm (SPEKOL 1300 UV VIS spectrophotometer, Analytik Jena, Jena, Germany). The ACC deaminase activity of the strains was measured with a calibration curve of α-ketobutyrate (5, 10, 15, 20, and 25 µmol mL^−1^), and results were reported as µmol α-ketobutyrate h^−1^ mg protein^−1^.

### 4.6. Molecular Identification and Phylogenetic Analysis

The most promising isolates (MTW 1 and MTW 5) were subjected to 16S rRNA gene barcoding by the Environmental Sciences Section, Department MeSVA, University of L’Aquila and BMR Genomics, Padua, Italy. A direct PCR was performed for DNA amplification using universal bacterial primers 27 F (5′-AGAGTTTGATCMTGG CTC AG-3′) and 1492 R (3′-TACGGYTACCTTGTTACG ACTT-5′). After combining the forward and reverse sequences, the consensus sequences obtained (~1400 bp) were compared to those available in the NCBI (National Centre for Biotechnology Information; http://www.ncbi.nlm.nih.gov/ accessed on 1 May 2025) genetic database. The aligned sequence produced was subjected to phylogenetic analysis, followed by a BLAST 2.17.0 with the nucleotide database of 16S ribosomal RNA sequences. MEGA software 11 was used to retrieve and analyse the most closely related species to the sequence. The phylogenetic tree was generated using the maximum likelihood approach. *Escherichia coli* U 5/41 (NR-024570.1) was utilised as an outgroup, and a maximum likelihood bootstrap analysis was carried out by reiterating the data matrix 1000 times (1000 bootstrap) [129,139].

### 4.7. In Planta Experiment on Lemna minor L.

Duckweed was sampled from different fountains in the Abruzzo region (Abruzzo, Italy). The macrophyte was placed in a tank with distilled water and oxygenated with an air pump for 15 days. The MTW 1 and MTW 5 strains showed good in vitro tolerance to heavy metals, and PGP activities were tested for compatibility before combining in a consortium for an in planta experiment on *Lemna minor* L. The consortium was adjusted at 10^6^ spores mL^−1^ as a final density. The inoculation was performed by immersing duckweed plants in the consortium for 1 h, while sterile distilled water was used for control plants. Based on the in vitro results, heavy metals (Al, Cd, Cu, Mn, Ni, Pb, and Zn) were combined in a mixture and applied for the in planta experiment [128]. The experiment was set up in glass containers for plants, and the experimental design was organised with nine repetitions per treatment as detailed below:

- CNT (without PGPB and heavy metals)
- PGPB (with PGPB and without heavy metals)
- CNT-HM (without PGPB and with heavy metals)
- PGPB-HM (with PGPB and heavy metals)

The experiment was performed for four weeks, after which the subsequent features were assessed: plant survival, plant biomass, root length, and the chlorophyll content [140].

### 4.8. Statistical Analysis

The data are presented as mean ± standard deviation, and each experiment was run in triplicate. The Shapiro–Wilk test was used to determine the data’s normality. A one-way analysis of variance (ANOVA) and Kruskal–Wallis test as a non-parametric test were performed with a 5% threshold of significance. All analyses were conducted using the XLSTAT 2016 software. Alpha-diversity measures and bar plots of ASV taxonomy, analysed at the phylum and genus levels, were produced using the Primer 7 and PAST 4.03 softwares.

## 5. Conclusions

Heavy metals contamination poses a substantial concern, owing to its negative environmental and human health impact. Compared to conventional methods, phytoremediation is ecologically friendly and cost-effective, requiring neither costly apparatus nor the specialised rehabilitation of polluted sites. Because of the amount of metal and mineral deposits and the enormous contamination that mines frequently produce, this study investigated the isolation of actinobacteria exhibiting tolerance to heavy metals and the ability to enhance plant development. The relevant strains were tested for their capacity to improve the phytoextraction capacity of *Lemna minor* L. The strains have been identified as *Streptomyces atratus*. The results demonstrated the strains’ capacity for heavy metal tolerance and several plant growth-promoting traits. These metal-tolerant actinobacteria have shown the ability to increase the phytoextraction capacity of duckweed by promoting plant development and photosynthesis under heavy metals stress. The experiment revealed the potential of utilising actinobacteria, specifically *Streptomyces*, as a biostimulant for phytoextracting plant species. This investigation demonstrates a viable environmentally friendly approach to bioremediation that uses microorganisms and plants with inherent resilience to enhance the clean-up of contaminated areas. Although the study was conducted under controlled conditions, and further research is needed to assess the effectiveness of this approach in diverse, real-world environments with varying levels of heavy metal contamination, these factors should be carefully considered in future studies to fully evaluate the feasibility of this bioremediation strategy. Further research is required to fully utilise this technique for the successful commercial application of cleaning polluted soil, and biotechnological technologies have been achieved in the engineering of plants for the improved phytoremediation of heavy metals.

## Figures and Tables

**Table 1:** Diversity indices obtained through the metabarcoding results of the 16S rRNA utilising PAST 4.03.

	LAK	MIN	MTW
Taxa_S	1273	1036	740
Individuals	28,505	18,070	13,029
Simpson_1-D	0.9978	0.998	0.9962
Shannon_H	6.635	6.577	6.134
Evenness_e^H/S^	0.5981	0.6935	0.6233
Chao-1	1274	1038	741.6

In the table, LAK: samples taken from the cave lake, MIN: innermost zone of the cave, and MTW: twilight zone near the inlet of the cave.

**Table 2:** Heavy metals tolerance of actinomycetes isolates evaluated on ISP_2_ agar medium.

	Al	Cd	Cu	Mn 0.5%	Mn 1%	Ni	Pb	Zn
MIN 1	+	-	++	-	-	+++	++	-
MIN 2	+	-	+++	-	-	+++	++	+++
MTW 1	+	+	+++	+++	+++	+++	++	+
MTW 2	+	+	++	+++	++	+++	+++	+
MTW 3	+	+	-	-	-	+	+	-
MTW 4	-	-	-	-	-	++	+	-
MTW 5	++	++	++	+++	++	+++	++	+
MTW 6	+	++	+	++	++	++	++	++
MTW 7	+	-	++	-	-	+++	+	-
MTW 8	+	+	++	-	-	+	++	+++

High tolerance (+++); moderate tolerance (++); low tolerance (+); and no tolerance (-).

**Table 3:** Plant growth-promoting traits of actinomycetes isolates.

	P µg mL^−1^	Ind µg mL^−1^	Sid %	HCN	NH_3_	ACCd
MIN 1	1.89 ± 0.00 ^a^	-	-	+	+++	-
MIN 2	-	-	13.15 ± 0.96 ^a^	+	+	-
MTW 1	13.37 ± 0.52 ^b^	2.85 ± 0.06	21.52 ± 2.16 ^a^	++	++	-
MTW 2	1.89 ± 0.00 ^a^	-	12.82 ± 0.76 ^a^	+	+	-
MTW 3	-	-	24.95 ± 1.25 ^a^	+	+	-
MTW 4	-	-	15.92 ± 2.30 ^a^	+	+	-
MTW 5	-	-	22.87 ± 3.06 ^a^	+	+++	-
MTW 6	-	-	16.70 ± 1.00 ^a^	+++	++	-
MTW 7	1.89 ± 0.00 ^a^	-	-	++	+++	-
MTW 8	-	-	-	++	++	-

Maximum activity (+++); moderate activity (++); low activity (+); and no activity (-). Results followed by identical case letters are not significantly different according to the Dunn post hoc test (phosphate) and Tukey’s post hoc test (siderophore) (*p* < 0.05) (*n* = 3). In the table, P: phosphate; Ind: indoles; Sid: siderophore; HCN: hydrogen cyanide; and NH_3_: ammonia, ACCd: Aminocyclopropane-1-Carboxylate Deaminase.

**Table 4:** Plant morpho-biometric parameters with and without heavy metals stress.

	Survival Rate (%)	Plant Biomass (%)	Root Lengths (cm)
CTL	100 ± 0 ^b^	2.26 ± 0.70 ^a^	2.37 ± 0.44 ^ab^
PGP	100 ± 0 ^b^	3.63 ± 0.33 ^a^	2.82 ± 0.08 ^ab^
CTL + HM	46.00 ± 0.82 ^ab^	1.87 ± 0.09 ^b^	1.45 ± 0.48 ^b^
PGP + HM	97.67 ± 0.47 ^ab^	2.33 ± 0.12 ^b^	1.77 ± 0.37 ^b^

Results followed by identical case letters are not significantly different according to the Dunn post hoc test (survival rate) and Tukey’s post hoc test (plant biomass and root lengths) (*p* < 0.05).

**Table 5:** The various heavy metals concentrations used for the in vitro HMs tolerance.

Heavy Metals	Final Concentration
Al_2_(SO_4_)_3_	2 mM
CdSO_4_·8H_2_O	0.1 mM
CuCl_2_·2H_2_O	0.15 mM
Pb(NO_3_)_2_	1 mM
NiCl_2_·6H_2_O	0.2 mM
ZnSO_4_·7H_2_O	1 mM

## Data Availability

Data are available from the corresponding author upon request. The data are not publicly available due to privacy and ethical restrictions.
